# Cube-based screening identifies a quinoa-derived synthetic microbial community that promotes plant growth and modulates root epidermal responses under salt stress

**DOI:** 10.3389/fpls.2026.1934646

**Published:** 2026-10-20

**Authors:** Hatairat Dangjarean, Yoshinori Murata, Yasufumi Kobayashi, Sara Neyrot, Takuya Ogata, Yasunari Fujita

**Affiliations:** 1 Graduate School of Science and Technology, University of Tsukuba, Tsukuba, Japan; 2 Japan International Research Center for Agricultural Sciences (JIRCAS), Tsukuba, Japan; 3 Rice Department, Ministry of Agriculture and Cooperatives (MOAC), Bangkok, Thailand; 4 Institute of Life and Environmental Sciences, University of Tsukuba, Tsukuba, Japan

**Keywords:** plant growth-promoting bacteria (PGPB), plant stress response, plant–microbe interaction, quinoa (*Chenopodium quinoa*), root epidermis, root hair, salt stress, synthetic microbial community (SynCom)

## Abstract

Plant-associated bacteria can improve plant performance under abiotic stress, but beneficial functions in plant microbiomes may depend on defined combinations of microorganisms rather than individual isolates alone. Here, we developed a cube-based screening strategy to identify functional synthetic microbial communities (SynComs) from 135 quinoa-associated bacterial isolates while preserving combinatorial diversity and traceability of isolate-level contributions. The isolates were divided into five 27-isolate sets, each arranged as a 3 × 3 × 3 cube in which each 3 × 3 layer was defined as a 9-isolate SynCom, generating 45 SynComs in total. Screening under 100 mM NaCl identified SynCom DY1 (SCDY1) as a candidate salt stress-mitigating consortium. SCDY1 consisted of nine taxonomically diverse isolates and exhibited a multifunctional profile, including siderophore production, phosphate solubilization, carboxymethyl cellulose degradation, indole compound production, and growth under saline conditions. In *Arabidopsis thaliana*, SCDY1 promoted primary root elongation in a salinity-dependent manner and increased biomass under moderate salinity conditions, with the clearest overall effects observed at 120 mM NaCl. At least a subset of the constituent bacteria was recoverable from inoculated seedlings. RNA sequencing and targeted RT-qPCR indicated that SCDY1 modulated host gene expression under moderate salinity stress, with responsive genes associated with oxidative stress, water- and oxygen-related processes, phenylpropanoid biosynthesis, glutathione metabolism, and root epidermis-related processes. Root hair phenotyping further showed that SCDY1 enhanced root hair-related traits and shifted visible root hair formation closer to the root apex. These findings identify a quinoa-derived SynCom that improves *Arabidopsis* performance under salinity stress and provide a practical, traceable framework for discovering beneficial microbial consortia from plant-associated bacterial collections.

## 1 Introduction

Soil salinity is a major environmental constraint limiting plant growth and agricultural productivity worldwide (Munns and Tester, 2008; Tang et al., 2024). Under saline conditions, plants are exposed to osmotic stress, ionic imbalance, nutrient disturbance, and oxidative stress, which collectively restrict root development, biomass accumulation, and yield (Hossain and Dietz, 2016; Munns and Tester, 2008). Because salinization is accelerated by irrigation, soil degradation, and climate change, sustainable strategies for improving plant performance under saline environments are increasingly needed (Tang et al., 2024). In addition to plant breeding and agronomic management, plant-associated microorganisms have attracted attention as biological resources that may help plants cope with abiotic stress.

Plant growth-promoting bacteria can influence host performance through diverse mechanisms, including phytohormone production, nutrient mobilization, siderophore production, modulation of stress responses, and alteration of root system architecture (Backer et al., 2018; Lugtenberg and Kamilova, 2009; Vacheron et al., 2013). Several bacterial isolates have been reported to promote plant growth under salt stress, indicating that plant-associated bacteria can contribute to stress adaptation through bacterial physiological functions as well as host transcriptional and physiological responses (Etesami and Glick, 2024; Yang et al., 2009). However, in natural plant-associated environments, bacteria rarely act as isolated entities. Instead, plant microbiomes consist of multiple interacting taxa whose collective functions may differ from those predicted from single-isolate traits alone (Trivedi et al., 2020). This complexity may partly explain why single-isolate inoculants can show context-dependent performance across plant species, stress intensities, and growth environments (Backer et al., 2018; Trivedi et al., 2020).

Synthetic microbial communities (SynComs) provide a powerful approach for studying and utilizing microbiome functions under controlled and reproducible conditions (Johns et al., 2016; Trivedi et al., 2020; Vorholt et al., 2017). Because SynComs are composed of defined microbial members, they offer greater reproducibility and interpretability than undefined microbial communities while retaining selected community-level properties. SynComs have been constructed using several strategies, including simplification of natural microbiomes, selection of representative or core community members (Niu et al., 2017; Trivedi et al., 2020; Vorholt et al., 2017), trait-based selection of functionally characterized isolates, and bottom-up assembly of defined microbial consortia (Johns et al., 2016). These approaches have advanced both plant microbiome research and broader synthetic community studies, but the design of effective SynComs remains challenging because community functions cannot always be predicted from individual isolate traits alone (Johns et al., 2016; Trivedi et al., 2020; Vorholt et al., 2017). In principle, combinatorial screening can reveal unexpected beneficial combinations; however, exhaustive testing rapidly becomes impractical as isolate number increases. Conversely, purely trait-based or fully rational designs may overlook beneficial interactions that emerge only in specific combinations (Johns et al., 2016; Vorholt et al., 2017). Thus, a practical challenge in SynCom-based approaches is how to preserve sufficient combinatorial diversity to discover unexpected community functions while retaining enough structure to infer whether beneficial activity is associated with dominant individual isolates or with specific combinations of multiple isolates. Previous studies using microbiomes derived from stress-adapted plants, including halophytes and desert plants, have demonstrated that selected microbial communities can improve the performance of non-host plants under salinity stress (Schmitz et al., 2022; Yuan et al., 2016). These studies support the idea that stress-adapted plants are valuable sources of beneficial microorganisms and highlight the need for screening strategies that can efficiently identify functional SynComs from large isolate collections. Plant-associated bacterial communities commonly include members of major phyla such as Pseudomonadota/Proteobacteria, Bacillota/Firmicutes, Actinomycetota/Actinobacteria, and Bacteroidota/Bacteroidetes (Choi et al., 2021). Therefore, SynCom screening strategies based on large isolate collections should ideally preserve broad taxonomic representation while remaining experimentally tractable.

Quinoa (*Chenopodium quinoa* Willd.) is a stress-resilient crop originating from the Andean region and is recognized for its broad adaptation to harsh environments, including saline and drought-prone conditions (Bazile et al., 2016; Hariadi et al., 2011). Recent genomic and transcriptomic studies have advanced our understanding of quinoa stress adaptation and salt tolerance, including genotype-dependent variation in salt-responsive growth traits, Na^+^ exclusion mechanisms, and the expansion of chromosome-scale genome resources across diverse quinoa accessions (Jarvis et al., 2017; Kobayashi et al., 2024, 2025; Mizuno et al., 2020; Rey et al., 2024, 2023; Yasui et al., 2016). In parallel, virus-induced gene silencing and virus-mediated overexpression systems have enabled functional analyses of quinoa genes, further strengthening quinoa as an emerging model for stress-resilient crop research (Ogata et al., 2021). Given its adaptation to stressful environments and the availability of genomic and functional resources, quinoa may represent a promising source of stress-associated microorganisms with potential plant growth-promoting functions. We previously identified a quinoa-associated bacterial isolate that promoted plant growth specifically under salinity stress conditions and exhibited salinity-responsive bacterial physiological traits (Murata et al., 2026). This single-isolate analysis suggested that quinoa-associated bacteria can contribute to plant–microbe interactions under salt stress. However, it remained unclear whether beneficial functions in quinoa-associated bacterial collections are primarily attributable to individual isolates or to combinations of multiple isolates. Importantly, our objective was not to further enhance the already high salinity tolerance of quinoa itself, but to determine whether microbial functions associated with this stress-adapted plant could confer measurable benefits in a more salt-sensitive glycophytic host. We therefore used *Arabidopsis thaliana* as a sensitive and experimentally tractable model system that enables reproducible and quantitative assessment of plant growth and host responses under controlled salinity conditions.

In the present study, we developed a cube-based SynCom screening strategy to identify salt stress-mitigating bacterial consortia from quinoa-associated isolates. This strategy was designed to preserve structured combinatorial diversity while keeping the number of SynComs within a practical screening scale. By arranging isolates in defined cubes, each isolate was included in multiple SynComs, allowing potential isolate-level contributions to remain traceable while testing different isolate combinations. Using this approach, we identified SynCom DY1 (SCDY1) as a quinoa-derived consortium that promotes *Arabidopsis* growth under moderate salinity stress. We further characterized its plant growth-promoting traits, recoverability from inoculated seedlings, and associated host transcriptional and root epidermal responses. Together, this study provides a practical framework for discovering beneficial SynComs from plant-associated bacterial collections and highlights quinoa-associated bacterial communities as promising resources of beneficial microorganisms under salinity stress.

## 2 Materials and methods

### 2.1 Isolation and taxonomic identification of quinoa-associated bacterial isolates

Endophytic bacteria were isolated from seeds and seedlings of quinoa (*Chenopodium quinoa* Willd.). The Kd inbred line (Yasui et al., 2016) was used as the main source material, and all seedling-derived isolates were obtained from Kd seedlings. Commercially available seeds derived from southern highland-type quinoa were also used for seed-borne bacterial isolation. The isolation procedure was performed as described previously (Murata et al., 2026), with minor modifications.

For isolation of seed-borne bacteria, quinoa seeds were surface-sterilized with 70% ethanol for 15–30 s or with a 1:100 dilution of Plant Preservative Mixture (Plant Cell Technology, Inc., Washington, DC, USA) for up to 5 min, followed by four rinses with sterile Milli-Q water. The sterilized seeds were imbibed in sterile Milli-Q water and placed on Reasoner’s 2A (R2A) agar plates or trypticase soy agar (TSA) plates. R2A agar plates were prepared with Difco R2A Agar at 18.2 g/L, and TSA plates were prepared with 3% (w/v) Trypticase Soy Broth and 1.8% (w/v) Difco Agar. All media components were purchased from Becton, Dickinson and Company (Franklin Lakes, NJ, USA). Plates were incubated at 25 °C for 2–3 days, and bacterial colonies emerging from the seeds were isolated and purified. To confirm surface sterilization, the final wash solution was spread on R2A agar plates and incubated at 25 °C.

For isolation of seedling-derived bacteria, quinoa seeds were surface-sterilized with 70% ethanol for 5–10 min, followed by immersion in 1.0% sodium hypochlorite solution for 5–10 min, and subsequently rinsed four times with sterile Milli-Q water. The sterilized seeds were placed on half-strength Murashige and Skoog (0.5× MS) agar medium supplemented with 3% (w/v) sucrose and incubated at 25 °C until germination. The medium was prepared with 0.5× MS salts (Fujifilm Wako Pure Chemical Corporation, Osaka, Japan) and 0.8% (w/v) Difco Agar (Becton, Dickinson and Company). Six days after germination, quinoa seedlings were transferred to cell trays containing a sterilized substrate composed of vermiculite and Potting Mix for Professionals (Innovex, Tokyo, Japan) at a ratio of 9:1 (v/v). The substrate had been autoclaved and dried before use. Seedlings were watered for one day and subsequently cultivated at 25 °C for 7 days under salt stress conditions with 300 mM NaCl.

After salt treatment, seedlings were surface-sterilized by washing three times with sterile Milli-Q water, followed by immersion in 70% ethanol for 1 min, immersion in 1.0% sodium hypochlorite solution for 1 min, and two additional washes with sterile Milli-Q water. To confirm the effectiveness of this final surface-sterilization step, the final rinse water was spread onto R2A agar plates and incubated at 25 °C for 3 days; no colony formation was observed. Residual moisture was removed using sterile paper towels. The sterilized tissues were homogenized with sterile zirconia beads at 1,100 rpm for 3 min using a Shake Master homogenizer (BMS-A20TP, BioMedical Science, Tokyo, Japan). The resulting homogenates were spread onto TSA and R2A agar plates and incubated at 25 °C for 4 days. Distinct bacterial colonies that appeared on the plates were isolated and purified.

Genomic DNA was extracted from each bacterial isolate using the Wizard Genomic DNA Purification Kit (Promega, Madison, WI, USA) according to the manufacturer’s instructions. Partial 16S rRNA gene sequences were amplified by PCR using MightyAmp DNA Polymerase Ver. 3.0 (Takara Bio Inc., Shiga, Japan) with the universal primers 27F and 1492R. PCR products were purified using the Wizard SV Gel and PCR Clean-Up System (Promega). The obtained sequences were compared with reference sequences using nucleotide BLAST searches, and taxonomic assignments were inferred based on the closest sequence similarities. A total of 135 quinoa-associated bacterial isolates were selected for SynCom construction. Based on partial 16S rRNA gene sequences, the 135 isolates were classified into four bacterial phyla: Bacillota (formerly Firmicutes; 57 isolates), Pseudomonadota (formerly Proteobacteria; 56 isolates), Actinomycetota (formerly Actinobacteria; 16 isolates), and Bacteroidota (formerly Bacteroidetes; 6 isolates). The taxonomic classification and cube-based allocation of the 135 isolates are listed in **Supplementary Table 1**. Isolate IDs indicate the original identification numbers assigned to the bacterial isolates.

### 2.2 Cube-based construction and screening of synthetic microbial communities

To construct defined synthetic microbial communities, the 135 bacterial isolates were divided into five independent sets of 27 isolates. The sets were assembled to include representatives of major plant-associated bacterial phyla and to maintain broad taxonomic diversity while avoiding overlap among isolates. During SynCom construction, isolates that did not grow reliably or could not be handled consistently under the experimental conditions were excluded or replaced. Although the taxonomic composition differed among the five sets, broad taxonomic representation was retained across the screening collection. Each 27-isolate set was arranged in a 3 × 3 × 3 cube. In each cube, every 3 × 3 layer along the x-, y-, and z-axes was defined as one 9-isolate SynCom, generating nine SynComs per cube and 45 SynComs in total. In this design, each isolate was included in three SynComs, one in each axial direction. The composition of the 45 SynComs generated by this cube-based design is listed in **Supplementary Table 2**.

For SynCom preparation, each constituent isolate was cultured in trypticase soy broth (TSB; 3% w/v) at 25 °C with shaking at 150 rpm, except for isolate 91, which was cultured at 91 rpm because it did not grow sufficiently at 150 rpm. To reduce the influence of differences in growth rate among isolates, bacterial cultures at different growth stages were combined before SynCom assembly. Each culture was adjusted to OD_600_ = 0.5, and the nine constituent isolates were mixed in equal volumes. The mixed bacterial suspension was washed at least three times with 10 mM MgCl_2_ to remove residual medium and then resuspended in 10 mM MgCl_2_. The final SynCom suspension was adjusted to OD_600_ = 0.025 unless otherwise stated. Mock treatment consisted of 10 mM MgCl_2_ without bacteria.

The 45 SynComs were screened for their ability to mitigate salt stress-associated growth inhibition in *Arabidopsis thaliana* Col-0 (CS60000). For primary screening, *Arabidopsis* seeds were sown on nylon mesh placed on the surface of rectangular agar plates. Nylon mesh sheets (NB34, nylon 66PA, 512-µm opening; Tokyo Screen Co., Ltd., Tokyo, Japan) were cut to 12 × 8 cm, soaked in 70% ethanol for 30 min, and UV-sterilized in a laminar flow cabinet for 30 min on each side. The sterilized mesh was placed on rectangular plates (10 × 14 cm) containing 65 mL of agar medium composed of 0.5× MS salts (Fujifilm Wako Pure Chemical Corporation, Osaka, Japan), 3% (w/v) sucrose, 0.05% (w/v) MES-KOH (pH 5.7), and 0.8% (w/v) agar. Sixty *Arabidopsis* seeds were sown on each mesh-covered plate. Plates were incubated horizontally at 22 °C under long-day conditions (16 h light/8 h dark) without cold stratification, and their positions were rotated daily to minimize positional effects.

At 5–6 days after sowing, seedlings were transferred to screening plates by moving the entire nylon mesh with sterile forceps. Screening plates were rectangular plates (10 × 14 cm) containing 65 mL of agar medium composed of 0.5× MS salts, 0% (w/v) sucrose, 0.05% (w/v) MES-KOH (pH 5.7), and 0.8% (w/v) agar, with or without 100 mM NaCl. Before transfer, 1 mL of SynCom suspension at OD_600_ = 0.025 or 10 mM MgCl_2_ mock solution was spread evenly over the surface of each screening plate using a sterile spreader and allowed to dry. In each experimental batch, nine SynComs and a 10 mM MgCl_2_ mock control were tested using two plates for each treatment × salinity combination, resulting in 40 screening plates per batch. Because inoculation and salinity treatments were applied at the plate level, the plate–mesh unit was considered the experimental unit, and individual seedlings within a plate were treated as subsamples. The 45 SynComs were evaluated across five experimental batches, and the entire five-batch screen was conducted twice, with the corresponding batches performed on separate dates using freshly prepared bacterial cultures, SynCom mixtures, and media, and independently grown plants.

All screening plates were photographed and assessed at the plate level. Because a complete, balanced quantitative dataset was not obtained for all 45 SynComs owing to occasional plate-level technical failures, the primary screen was treated as an exploratory candidate-selection step. Candidates were prioritized by comparison with the corresponding within-batch mock control under both salinity conditions and by the consistency of the response across the two screening rounds. Eleven SynComs were shortlisted for further image-based leaf area assessment using ImageJ/Fiji. Among these candidates, SCDY1 showed the most consistent response across the two rounds and was selected for subsequent quantitative validation as SCDY1. The closest related taxa and sequence-based taxonomic information for the SCDY1 constituent isolates are listed in **Supplementary Table 3**.

### 2.3 Phylogenetic analysis of SCDY1 constituent isolates

The nine bacterial isolates constituting SCDY1 were subjected to phylogenetic analysis based on partial 16S rRNA gene sequences. Sequences of SCDY1 constituent isolates and closely related reference taxa were aligned using MUSCLE. A phylogenetic tree was constructed using the neighbor-joining method with 1,000 bootstrap replicates. The resulting tree was used to examine the taxonomic relationships of the nine SCDY1 constituent isolates.

### 2.4 Characterization of plant growth-promoting traits and bacterial growth under saline conditions

Plant growth-promoting traits of SCDY1 and its individual constituent isolates were characterized using plate-based and colorimetric assays. The tested traits included siderophore production, phosphate solubilization, carboxymethyl cellulose degradation, indole compound production, and bacterial growth under saline conditions. For plate-based assays, bacterial suspensions of SCDY1 or each individual constituent isolate were prepared in 10 mM MgCl_2_ and adjusted to the indicated optical density. Aliquots of 3–4 µL were spotted onto the corresponding assay medium and incubated at room temperature. Plates were scanned using a flatbed scanner, and colony diameters, halo diameters, or activity zones were quantified using ImageJ/Fiji.

Siderophore production was evaluated using chrome azurol S (CAS) agar (Louden et al., 2011; Murata et al., 2026), with minor modifications. Bacterial suspensions were spotted onto CAS agar plates, and siderophore production was assessed based on the formation of orange halo zones around bacterial colonies. Thirteen days after inoculation, the diameter of the surrounding orange halo was measured. Phosphate solubilization activity was evaluated using phosphate agar medium containing tricalcium phosphate as an insoluble phosphate source (Chen and Liu, 2019; Murata et al., 2026), with minor modifications. Bacterial suspensions were spotted onto phosphate agar plates and incubated for 13 days. Phosphate solubilization activity was evaluated by measuring the clear halo diameter surrounding bacterial colonies. Carboxymethyl cellulose (CMC) degradation activity was evaluated using CMC-Na agar medium (Liang et al., 2014; Murata et al., 2026), with minor modifications. Bacterial suspensions were spotted onto CMC-Na agar plates and incubated for 7 days. Plates were subsequently stained with 0.1% (w/v) Congo-red solution, and the clear halo diameter surrounding bacterial colonies was measured as an indicator of CMC degradation activity. Indole compound production was quantified using the Salkowski reagent method (Gordon and Weber, 1951). Bacterial cultures were grown in 1/10-strength TSB supplemented with 1 mM L-tryptophan, and culture supernatants were mixed with Salkowski reagent. After incubation in the dark at room temperature, absorbance was measured at 530 nm. Indole compound concentration was estimated using a standard curve prepared with indole-3-acetic acid. Bacterial growth under saline conditions was evaluated by monitoring optical density at 600 nm over 50 h in culture medium supplemented with 0, 100, 120, or 150 mM NaCl. The NaCl concentrations used for the bacterial growth assay were selected to cover the salinity range used across the plant assays, including 100, 120, and 150 mM NaCl. Multifunctional profiles of SCDY1 and its constituent isolates were visualized using radar plots generated with the fmsb package in R.

### 2.5 *Arabidopsis* growth assay under salinity stress

*Arabidopsis thaliana* Col-0 seeds were surface-sterilized and stratified at 4 °C for 48 h to synchronize germination (Nagatoshi et al., 2023). Seeds were germinated on modified 0.5× MS medium containing 3% sucrose and agar. Seedlings were grown vertically in a controlled growth chamber at 22 °C under long-day conditions with white light at an intensity of 45 µmol m^-^² s^-^¹ and 60% relative humidity. Uniformly sized seedlings were transferred to 0.5× MS medium without sucrose and supplemented with or without NaCl. For the initial growth assay, seedlings were transferred to medium containing 0 or 100 mM NaCl. For each treatment × salinity combination, three replicate plates were used, each containing eight individually inoculated seedlings (24 seedlings per combination). After acclimation, *Arabidopsis* seedlings were inoculated with 4 µL of SCDY1 suspension adjusted to OD_600_ = 0.025 near the root tip region. A 10 mM MgCl_2_ solution was applied in the same manner as the mock control. Plates were maintained vertically, and their positions were rotated daily to minimize positional effects. Primary root length was monitored over time by marking root tips and scanning plates with a flatbed scanner. Root length was quantified from scanned images using ImageJ/Fiji. At 187 h after inoculation, the time point used for the treatment-by-salinity interaction analysis, none of the roots had reached the bottom edge of the plates. Fresh and dry weights were measured for individual seedlings, with fresh weight determined immediately after harvest and dry weight determined after drying at 60 °C until a constant weight was obtained.

To further evaluate the salinity range of SCDY1 activity, additional assays were conducted under 0, 120, and 150 mM NaCl conditions. Seedlings with similar primary root lengths were transferred to the corresponding salinity media and acclimated before inoculation. In each experiment, each treatment × salinity combination was represented by one plate containing six individually inoculated seedlings, with the mock and SCDY1 treatments maintained on separate plates. SCDY1 suspension or 10 mM MgCl_2_ mock solution was applied to each seedling, and the plants were grown vertically under controlled conditions. Primary root length, fresh weight, and dry weight were measured for individual seedlings. The experiment was independently conducted three times on separate dates.

### 2.6 Re-isolation of SCDY1-derived bacteria from inoculated seedlings

To examine whether SCDY1-derived bacteria could be recovered from inoculated plants, *Arabidopsis thaliana* seedlings were transferred to 0.5× MS medium supplemented with 100, 120, or 150 mM NaCl (Murata et al., 2026), with minor modifications. One day after transfer, seedlings were inoculated with 4 µL of SCDY1 suspension adjusted to OD_600_ = 0.025 or with 4 µL of 10 mM MgCl_2_ as the mock control. Six days after inoculation, plant tissues were homogenized with sterile zirconia beads at 1,100 rpm for 3 min using a Shake Master homogenizer, and serial dilutions of the homogenates were spread onto tryptic soy agar, one-tenth-strength tryptic soy agar, and R2A agar plates. Bacterial colonies recovered from SCDY1-inoculated seedlings were subjected to colony PCR and sequence analysis for taxonomic identification. The re-isolation experiment was performed twice independently.

### 2.7 RNA extraction and RNA-seq analysis

For transcriptome analysis, *Arabidopsis* seedlings were grown under four treatment conditions: mock-treated seedlings under non-saline conditions, mock-treated seedlings under 120 mM NaCl, SCDY1-treated seedlings under non-saline conditions, and SCDY1-treated seedlings under 120 mM NaCl. These groups were designated M0, M120, S0, and S120, respectively. SCDY1 or mock solution was applied to seedlings, and plant samples were collected at 100 h after inoculation, when SCDY1-dependent growth differences were detectable under 120 mM NaCl. Four biological replicates were prepared for each treatment, with each replicate consisting of pooled seedlings.

Total RNA was extracted using RNAiso Plus (Takara Bio Inc., Shiga, Japan) according to the manufacturer’s protocol (Nagatoshi et al., 2023), with minor modifications. RNA quality and quantity were assessed before library preparation. RNA-seq libraries were constructed and sequenced using the Illumina NovaSeq platform (Illumina Inc., San Diego, CA, USA). Raw sequencing reads were quality-filtered to remove adapter sequences and low-quality bases using Trimmomatic v0.41 (Bolger et al., 2014). Cleaned reads were aligned to the *Arabidopsis thaliana* TAIR10 reference genome using HISAT2 v2.1.0 (Kim et al., 2019). Gene-level read counts were quantified using featureCounts (Liao et al., 2014), and gene expression levels were calculated as transcripts per million (TPM). Differentially expressed genes were identified using edgeR (Robinson et al., 2010). Four pairwise comparisons were analyzed: M120 vs M0, S120 vs S0, S0 vs M0, and S120 vs M120. Genes with an absolute log_2_ fold change of at least 0.585 and a false discovery rate below 0.05 were defined as differentially expressed genes. To test whether the SCDY1 response depended on salinity, gene-wise generalized linear models including SCDY1 inoculation, salinity, and their interaction were fitted using edgeR. Interaction terms were evaluated by likelihood ratio tests, with FDR < 0.05 defining significance. Separately, DEGs identified in at least one pairwise comparison were combined, their TPM values z-scaled, and hierarchically clustered across M0, S0, M120, and S120 using heatmap.2 in the gplots package for descriptive visualization rather than interaction testing. DEG distributions were visualized using bar plots and volcano plots. Principal component analysis was performed using log_2_-transformed expression values. Overlaps among DEG sets were visualized using Venn diagrams.

### 2.8 Functional enrichment analysis

Gene Ontology (GO) enrichment analyses were performed for two DEG sets derived from the pairwise comparisons. The 135 DEGs unique to S120 vs M120 among the four pairwise DEG lists were analyzed for biological process enrichment, whereas all 567 DEGs identified in S120 vs M120 were analyzed for both biological process and molecular function enrichment. Genes identified by the gene-wise interaction analysis were not included in either GO analysis. Enrichment was analyzed using clusterProfiler (Yu et al., 2012) with org.At.tair.db and Benjamini–Hochberg-adjusted *p* values (Benjamini and Hochberg, 1995). Closely related or hierarchically overlapping GO terms were summarized as broader biological themes. KEGG pathway enrichment analysis was performed using DEGs identified in the S120 vs M120 comparison to identify metabolic and signaling pathways associated with SCDY1-responsive transcriptional changes under salinity stress (Kanehisa and Goto, 2000). Enriched KEGG pathways were identified using adjusted *p* values.

### 2.9 RT-qPCR analysis

To confirm selected RNA-seq expression patterns using a targeted quantification method, RT-qPCR analysis was performed using the same RNA samples that were used for RNA-seq analysis from mock-treated and SCDY1-treated *Arabidopsis* seedlings grown under 120 mM NaCl. Total RNA was extracted from plant tissues using RNAiso Plus. For first-strand cDNA synthesis, 1 µg of total RNA was treated with RQ1 RNase-Free DNase (Promega, Madison, WI, USA) for 30 min at 37 °C, and cDNA was synthesized using the SuperScript III First-Strand Synthesis System (Invitrogen, Carlsbad, CA, USA) according to the manufacturer’s instructions. RT-qPCR was performed on a QuantStudio 7 Flex Real-Time PCR System (Applied Biosystems, Foster City, CA, USA) using GoTaq qPCR Master Mix (Promega) and gene-specific primers for selected phenylpropanoid-, root-associated-, and redox-related genes. The primers used in this study are listed in **Supplementary Table 4**. Gene expression levels were normalized using *PP2A* as the reference gene. Relative expression levels were calculated against the mean expression level of the mock-treated seedlings under 120 mM NaCl (M120), which was set to 1 for each gene.

### 2.10 Root hair analysis

Root hair phenotypes were analyzed in mock-treated and SCDY1-treated *Arabidopsis* seedlings grown under 0 or 120 mM NaCl. Five-day-old seedlings grown on 0.5× MS medium with or without 120 mM NaCl were inoculated with mock solution or SCDY1. Root hair phenotypes were analyzed 48 h after inoculation, corresponding to 7-day-old seedlings. The primary root and mature root hair region were observed using a Leica M205 FA stereomicroscope equipped with a Leica DFC7000 T camera (Leica Microsystems, Wetzlar, Germany). Primary root length was measured from root images using ImageJ/Fiji. Root hair zone length was measured as the length of the root hair-bearing region formed after inoculation. The distance from the root apex to the first visible root hair was measured as an index of the position of root hair differentiation. Root hair density and root hair length were quantified in a 3-mm root segment located 2 mm from the primary root tip. Root hair density was calculated as the number of root hairs per millimeter of primary root length. Root hair length was measured for all visible root hairs within the same 3-mm root segment.

### 2.11 Statistical analysis

Statistical analyses and data visualization were performed using R (R Core Team, 2024). The primary SynCom screen was evaluated descriptively and used for exploratory candidate selection. The plate–mesh unit was defined as the experimental unit, with individual seedlings within each plate treated as subsamples. Formal statistical inference concerning SCDY1 was based on the subsequent quantitative validation experiments. For the initial growth assay, individual-seedling data were used for within-salinity comparisons. For the interaction analysis at 187 h, root-length measurements were averaged across the eight seedlings on each plate, and the resulting plate means (*n* = 3 per treatment × salinity combination) were analyzed by two-way ANOVA with SCDY1 treatment, NaCl concentration, and their interaction, followed by Tukey’s HSD test. The salinity-range experiment was analyzed by one-way ANOVA followed by Tukey’s HSD test; other two-group comparisons were performed using Student’s *t*-test. For RT-qPCR analysis, statistical significance between mock-treated and SCDY1-treated seedlings under 120 mM NaCl was assessed using Student’s *t*-test. Differences were considered statistically significant at *p* < 0.05.

## 3 Results

### 3.1 Cube-based screening of quinoa-derived SynComs identifies SynCom DY1 as a candidate salt stress-mitigating consortium

To extend our previous single-isolate analysis of quinoa-associated bacteria (Murata et al., 2026), we designed a cube-based SynCom screening strategy to examine whether salt stress-mitigating activity is primarily associated with individual isolates or with combinations of multiple isolates (**Figure 1**). A total of 135 bacterial isolates obtained from quinoa seeds and seedlings were used for SynCom construction. Based on partial 16S rRNA gene sequences, the 135 isolates were classified into four bacterial phyla: Bacillota (formerly Firmicutes; 57 isolates), Pseudomonadota (formerly Proteobacteria; 56 isolates), Actinomycetota (formerly Actinobacteria; 16 isolates), and Bacteroidota (formerly Bacteroidetes; 6 isolates) (**Figure 1A**; **Supplementary Table 1**). Although the taxonomic composition differed among the five sets, the cube-based design retained broad representation of multiple major plant-associated bacterial phyla across the screening collection. Each 27-isolate set was arranged in a 3 × 3 × 3 cube, and each 3 × 3 layer along the x-, y-, and z-axes was defined as a 9-isolate SynCom (**Figure 1B**). This design generated nine SynComs per cube and 45 SynComs in total (**Figure 1A**). An important feature of this design is that each isolate is included in three SynComs, one in each axial direction (**Figure 1B**). Therefore, activity shared across three SynComs could indicate a dominant isolate-level contribution, whereas layer-specific activity could suggest a combination-dependent effect. This design enabled structured screening of quinoa-derived bacterial communities while retaining traceability of isolate-level and community-level contributions.

**Figure 1:**
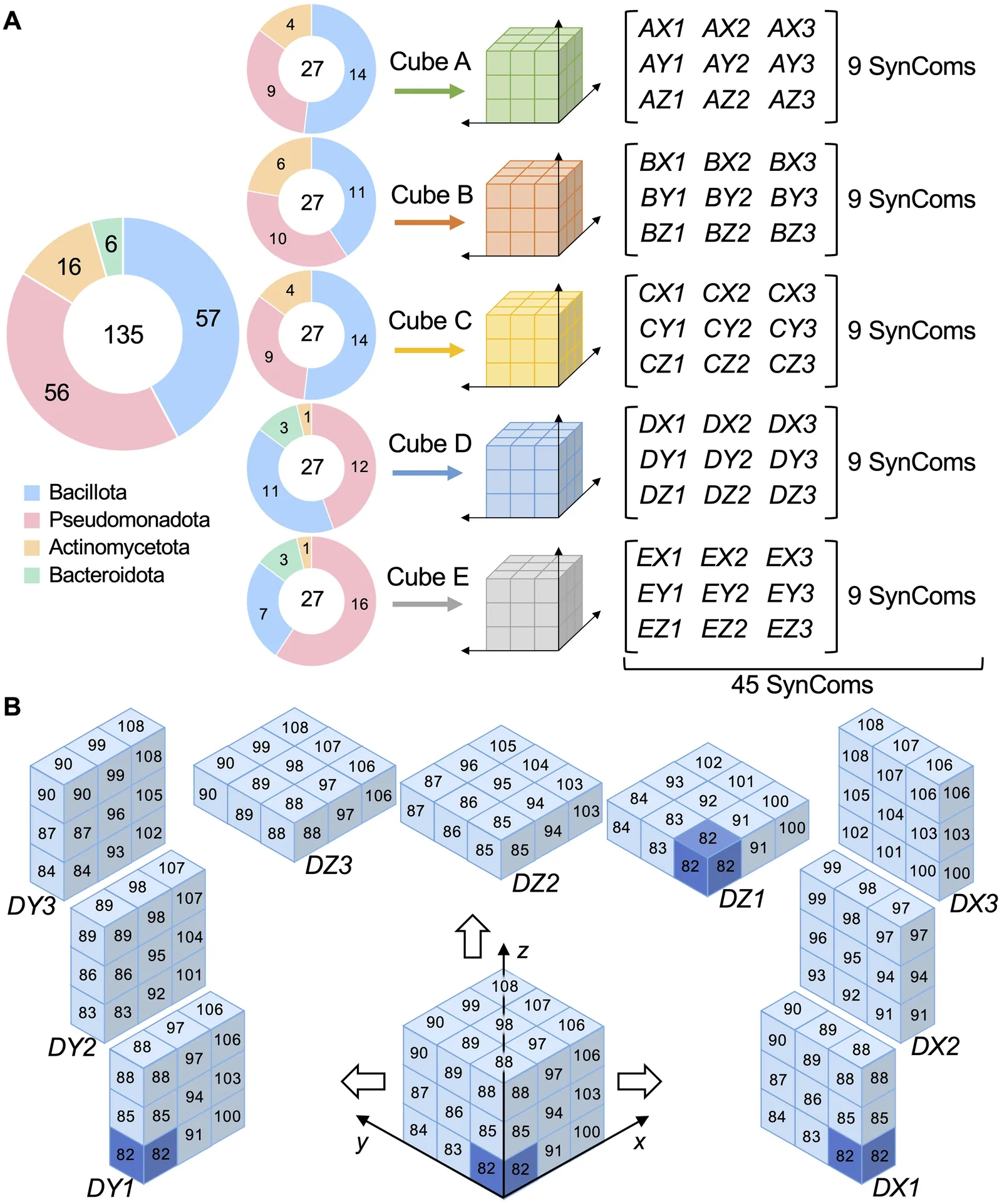
Cube-based screening strategy for identifying quinoa-derived SynComs with salt stress-mitigating activity. **(A)** Schematic overview of the cube-based SynCom screening design. A total of 135 quinoa-associated bacterial isolates were divided into five independent 27-isolate sets, designated Cubes A–E. The sets were assembled to retain broad taxonomic diversity and to include representatives of major plant-associated bacterial phyla while avoiding overlap among isolates. Each 27-isolate set generated nine 9-isolate SynComs, resulting in 45 SynComs in total. **(B)** Schematic representation of the layer-based definition of SynComs within a representative 3 × 3 × 3 cube. Each 3 × 3 layer along the x-, y-, and z-axes was defined as one 9-isolate SynCom, so that each isolate was included in three SynComs, one in each axial direction. SynCom DY1 consisted of nine constituent isolates with isolate IDs 82, 85, 88, 91, 94, 97, 100, 103, and 106. Isolate 82 was included in SynComs DY1, DX1, and DZ1, illustrating how each isolate was represented in three different community contexts. Among the SynComs evaluated in the exploratory primary screen, DY1 showed the most consistent candidate response across the two screening rounds and was therefore prioritized for subsequent quantitative validation. SynCom DY1 (SCDY1) was selected for subsequent analyses.

The 45 SynComs were evaluated in two exploratory screening rounds. Based on the technically evaluable plates, 11 SynComs showed candidate responses characterized by improved seedling growth relative to the corresponding within-batch mock control under 100 mM NaCl, with little or no apparent effect under non-saline conditions. SynCom DY1 (SCDY1) showed the most consistent candidate response across the two rounds and was therefore selected for subsequent quantitative validation (**Supplementary Table 2**). SCDY1 was composed of nine predefined quinoa-associated bacterial isolates that had been assigned to the SynCom DY1 layer during the cube-based screening design (**Figure 1B**). Based on partial 16S rRNA gene sequences, the nine SCDY1 constituent isolates were closely related to *Pseudomonas panipatensis* (isolate 82), *Stenotrophomonas bentonitica* (isolate 85), *Fictibacillus enclensis* (isolate 88), *Novosphingobium subterraneum* (isolate 91), *Herbaspirillum frisingense* (isolate 94), *Acidovorax facilis* (isolate 97), *Bacillus atrophaeus* (isolate 100), *Priestia megaterium* (isolate 103), and *Bacillus subtilis* (isolate 106) (**Figure 2A** and **Supplementary Table 3**). These isolates belonged to five bacterial orders, Pseudomonadales, Xanthomonadales, Sphingomonadales, Burkholderiales, and Bacillales, indicating that SCDY1 represented a taxonomically diverse 9-isolate SynCom selected through the cube-based screening strategy.

**Figure 2:**
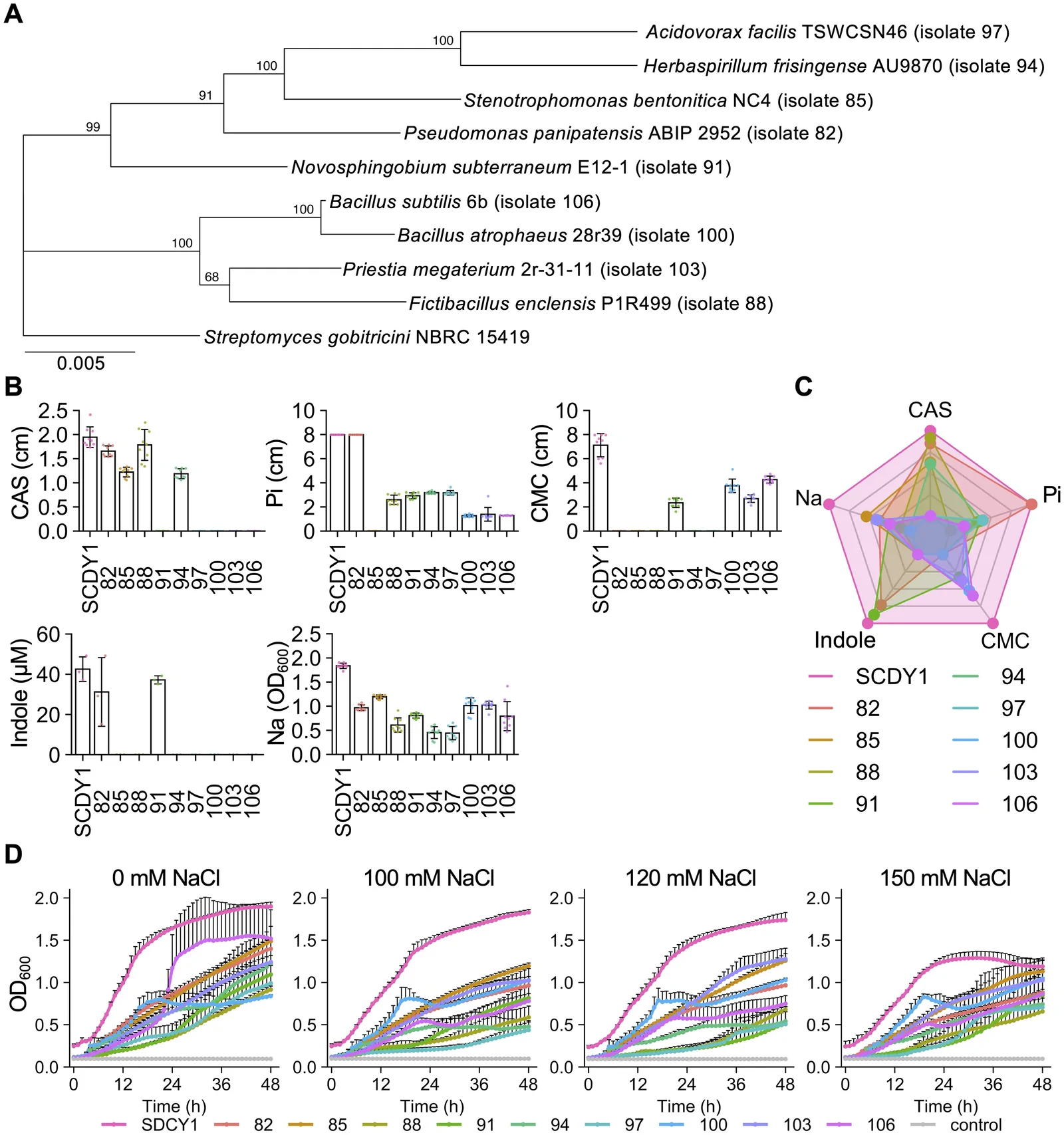
Taxonomic diversity and functional traits of SCDY1. **(A)** Phylogenetic tree of the nine SCDY1 member isolates based on partial 16S rRNA gene sequences. The isolates were closely related to *Pseudomonas panipatensis* (isolate 82), *Stenotrophomonas bentonitica* (isolate 85), *Fictibacillus enclensis* (isolate 88), *Novosphingobium subterraneum* (isolate 91), *Herbaspirillum frisingense* (isolate 94), *Acidovorax facilis* (isolate 97), *Bacillus atrophaeus* (isolate 100), *Priestia megaterium* (isolate 103), and *Bacillus subtilis* (isolate 106). *Streptomyces gobitricini* NBRC 15419 was used as an outgroup. Numbers at nodes indicate bootstrap values, and the scale bar indicates substitutions per site. **(B)** Plant growth-promoting traits of SCDY1 and its constituent isolates, including siderophore production, CMC degradation, phosphate solubilization, indole compound production, and growth under saline conditions (labeled CAS, CMC, Pi, Indole, and Na, respectively, in **Figure 2**). Bars represent mean values, and error bars indicate standard deviation. **(C)** Radar chart comparing the multifunctional profiles of SCDY1 and the individual member isolates. **(D)** Growth curves of SCDY1 and individual isolates in media supplemented with 0, 100, 120, or 150 mM NaCl. Bacterial growth was monitored as OD_600_ over 50 h. Values represent mean ± SD.

### 3.2 SCDY1 exhibits a multifunctional profile of plant growth-promoting traits

Having selected SCDY1 through the cube-based screening strategy, we next examined whether this 9-isolate SynCom possessed plant growth-promoting traits that could contribute to salt stress mitigation. We evaluated siderophore production, phosphate solubilization, carboxymethyl cellulose (CMC) degradation, indole compound production, and bacterial growth under saline conditions for SCDY1 and its constituent isolates. The SCDY1 member isolates displayed distinct functional profiles across the tested plant growth-promoting traits (**Figure 2B**; **Supplementary Table 5**). Among the individual isolates, isolate 88 showed the highest siderophore production. Isolate 82 showed the highest phosphate solubilization activity among the individual isolates, reaching a level comparable to that of SCDY1. Isolate 106 showed the highest CMC degradation activity among the individual isolates, corresponding to 60% of the SCDY1 value. Isolate 91 exhibited the highest indole compound production among the individual isolates, corresponding to 87.5% of the SCDY1 value. In addition, isolate 85 showed the highest growth under saline conditions among the individual isolates, corresponding to 65.0% of the SCDY1 value. These results indicate that different member isolates of SCDY1 contributed distinct functional capacities rather than uniformly showing the same plant growth-promoting traits. When the multifunctional profiles of the member isolates and SCDY1 were compared by radar chart analysis, SCDY1 showed a broad activity profile across the tested traits (**Figure 2C**). Except for phosphate solubilization by isolate 82, SCDY1 exceeded the individual member isolates in all tested plant growth-promoting traits (**Figure 2C**; **Supplementary Table 5**). Thus, SCDY1 exhibited a broad multifunctional profile rather than a single dominant plant growth-promoting trait, consistent with the distribution of distinct functional capacities among its member isolates.

We further examined bacterial growth under saline conditions by monitoring optical density over 50 h in media containing 0, 100, 120, or 150 mM NaCl. Across all NaCl concentrations tested, SCDY1 showed faster growth than each of its individual constituent isolates (**Figure 2D**). Together, these results show that SCDY1 combines multifunctional plant growth-promoting traits with robust growth under saline conditions.

### 3.3 SCDY1 promotes *Arabidopsis* growth under moderate salinity conditions

To determine whether the selected consortium could mitigate salt stress-associated growth inhibition in plants, we examined the effects of SCDY1 inoculation on *Arabidopsis* seedlings grown under non-saline and saline conditions. *Arabidopsis* was used as a sensitive and experimentally tractable glycophytic model host to quantitatively evaluate root growth and biomass responses to microbial inoculation under controlled salinity stress. Seedlings were transferred to medium with or without NaCl and inoculated with SCDY1 or mock solution near the root tip region (**Figure 3A**). Under non-saline conditions, SCDY1 did not promote primary root elongation (**Figure 3B**), and fresh and dry weights were comparable between treatments (**Figures 3C, D**). In contrast, under 100 mM NaCl, SCDY1 enhanced primary root elongation (**Figures 3A, E**) and increased fresh and dry weights (**Figures 3F, G**). At 187 h, when none of the roots had reached the bottom edge of the plates, two-way ANOVA of plate means (*n* = 3 plates per treatment × salinity combination) detected a significant SCDY1 treatment × NaCl interaction for primary root length (F(1, 8) = 41.18, *p* = 2.05 × 10^-4^; **Figure 3H**). Tukey’s HSD test showed that SCDY1 slightly reduced primary root length under non-saline conditions (*p* = 0.016) but increased it under 100 mM NaCl (*p* = 0.004), providing direct statistical support for a salinity-dependent effect of SCDY1 on primary root elongation.

**Figure 3:**
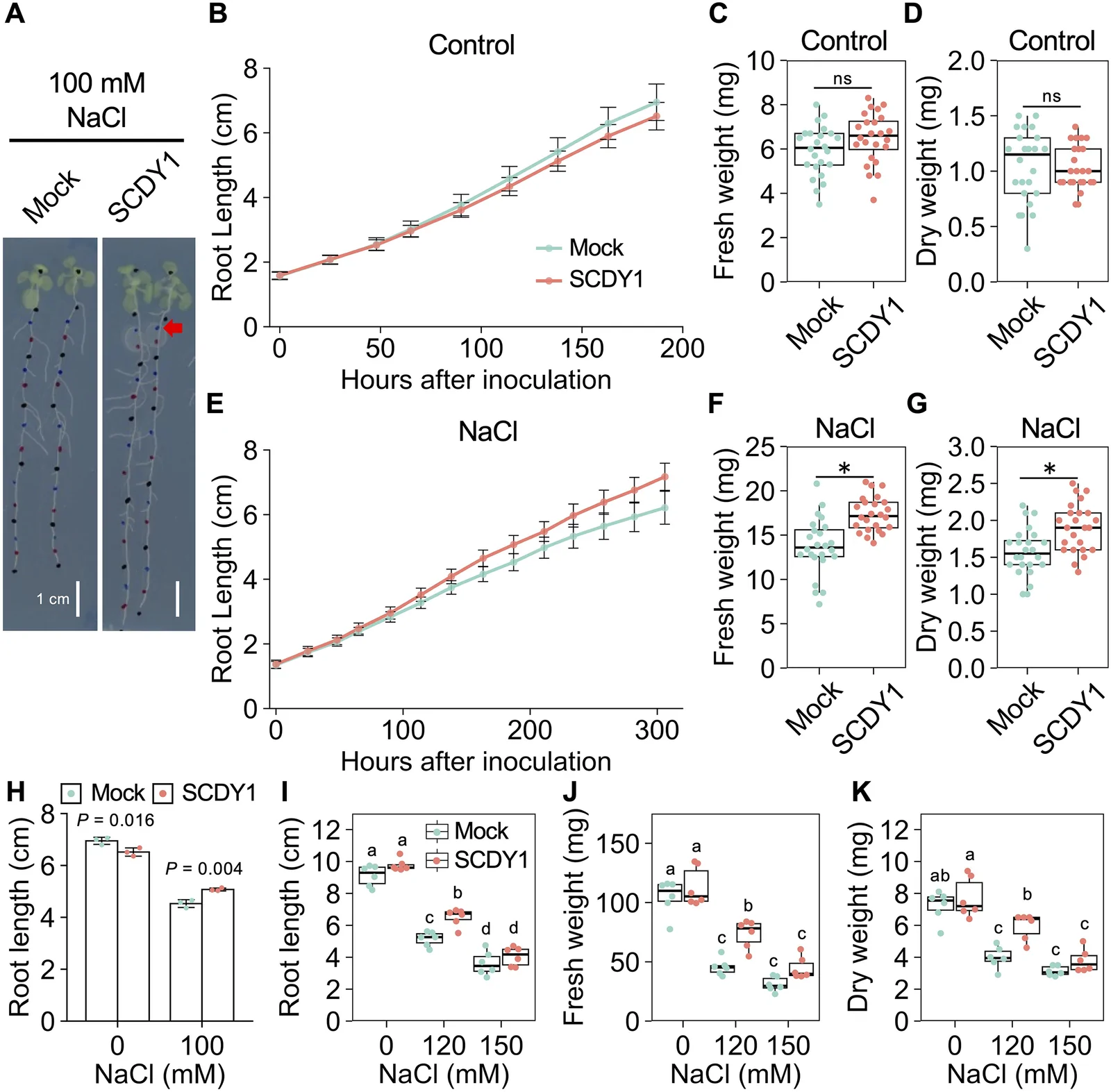
SCDY1 promotes *Arabidopsis* seedling growth under moderate salinity conditions. **(A)** Representative images of mock-treated and SCDY1-treated seedlings grown under 100 mM NaCl. The arrow indicates the SCDY1 inoculation point near the root tip, and colored marks indicate sequential root-tip positions used to monitor primary root elongation. Scale bars = 1 cm. **(B, E)** Time-course analysis of primary root elongation under non-saline conditions **(B)** and 100 mM NaCl **(E)**. **(C, D, F, G)** Fresh weight **(C, F)** and dry weight **(D, G)** under non-saline conditions **(C, D)** and 100 mM NaCl **(F, G)**. For **(B–G)**, 24 individually inoculated seedlings from three replicate plates were analyzed per treatment × salinity combination. Lines in **(B, E)** show mean ± SD, and box plots in **(C, D, F, G)** show medians, interquartile ranges, whiskers, and individual seedlings. In **(C, D, F, G)**, “ns” indicates no significant difference, and an asterisk indicates *p* < 0.05, as determined by Student’s *t*-test. Individual seedlings in **(B–G)** were subsamples within plates. The treatment × salinity interaction in primary root length was evaluated using plate means in panel **(H)**. **(H)** Plate-level analysis of primary root length at 187 h after inoculation under 0 and 100 mM NaCl. Each dot represents the mean primary root length of eight seedlings on one plate (*n* = 3 plates per treatment × salinity combination), and bars show mean ± SD of the three plate means. Two-way ANOVA detected a significant SCDY1 treatment × NaCl interaction (F(1, 8) = 41.18, *p* = 2.05 × 10^-4^). The indicated *p* values were obtained using Tukey’s HSD test. **(I–K)** Primary root length **(I)**, fresh weight **(J)**, and dry weight **(K)** of mock-treated and SCDY1-treated seedlings grown under 0, 120, or 150 mM NaCl. Each dot represents one individually inoculated seedling (*n* = 6 seedlings from one plate per treatment × salinity combination). One representative experiment of three independently conducted experiments with similar overall trends is shown. Box plots show medians, interquartile ranges, whiskers, and individual seedlings. Different lowercase letters summarize comparisons of individual-seedling values within the representative experiment (*p* < 0.05, one-way ANOVA followed by Tukey’s HSD test). Panels I–K provide a descriptive view of the response pattern observed in the representative experiment.

To further examine the effects of SCDY1 across salinity levels, *Arabidopsis* seedlings were exposed to 0, 120, or 150 mM NaCl. Across three independently conducted experiments, the overall growth-response pattern was similar, although the magnitude of the response varied among experiments. In the representative experiment shown in **Figures 3I–K**, SCDY1-treated seedlings had greater primary root length, fresh weight, and dry weight under 120 mM NaCl, whereas comparable increases were not observed under 0 or 150 mM NaCl. **Figures 3I–K** present the individual-seedling responses observed in one representative experiment and provide a descriptive view of the response pattern. Accordingly, 120 mM NaCl, at which the SCDY1-associated growth response was most clearly observed, was selected for subsequent transcriptome analysis of host responses associated with SCDY1 treatment under moderate salinity conditions.

To confirm that SCDY1-derived bacteria were present in the plant assay system after inoculation, we performed a culture-dependent re-isolation assay. Seedlings inoculated with SCDY1 or mock solution were homogenized after growth under salinity stress, and the homogenates were plated on bacterial culture media. Bacterial colonies were recovered from SCDY1-inoculated seedlings on multiple media, whereas no colonies were obtained from mock-treated seedlings. Colony PCR followed by sequence analysis identified colonies corresponding to several SCDY1 constituent isolates, including isolates 82, 85, 94, and 106 (**Supplementary Table 6**). These results indicate that at least a subset of SCDY1 constituent bacteria was recoverable from inoculated seedlings under salinity stress conditions.

### 3.4 SCDY1 modulates the *Arabidopsis* salt stress transcriptome under moderate salinity stress

Because SCDY1 promoted *Arabidopsis* growth most clearly under 120 mM NaCl in our phenotypic assay, this condition was used as a moderate salinity stress condition for transcriptome analysis. Total RNA was extracted from seedlings at 100 h after inoculation, when SCDY1-dependent differences in growth phenotype were clearly detectable under 120 mM NaCl. Four treatment groups were analyzed: mock-treated seedlings under non-saline conditions (M0), mock-treated seedlings under 120 mM NaCl (M120), SCDY1-treated seedlings under non-saline conditions (S0), and SCDY1-treated seedlings under 120 mM NaCl (S120). Differentially expressed genes (DEGs) were identified using four pairwise comparisons: M120 vs M0, S120 vs S0, S0 vs M0, and S120 vs M120. Salt stress caused the largest transcriptional changes in both mock- and SCDY1-treated plants, with 2,256 DEGs in M120 vs M0 and 1,778 DEGs in S120 vs S0 (**Figure 4A**; **Supplementary Table 7**). Direct comparisons between SCDY1- and mock-treated plants identified fewer DEGs, with 341 DEGs under non-saline conditions and 567 DEGs under 120 mM NaCl conditions. The direction of SCDY1-responsive transcriptional changes differed between non-saline and saline conditions. Under non-saline conditions, most SCDY1-responsive DEGs were upregulated, with 299 upregulated and 42 downregulated genes in S0 vs M0. In contrast, under 120 mM NaCl, SCDY1-responsive DEGs included similar numbers of upregulated and downregulated genes, with 256 upregulated and 311 downregulated genes in S120 vs M120. These results indicate that salt stress was the major driver of transcriptional variation, whereas SCDY1 induced a distinct transcriptional response that was smaller than the salinity-driven response. The balanced up- and downregulation observed under 120 mM NaCl suggests that SCDY1 modulated, rather than simply activated, the host salt stress response. Volcano plot analysis further showed that many SCDY1-responsive DEGs displayed moderate fold changes rather than large expression shifts (**Figure 4B**). Principal component analysis (PCA) showed that PC1, which explained 45.6% of the total variance, separated samples primarily according to salinity treatment, whereas PC2, which explained 10.9% of the variance, reflected variation associated with SCDY1 treatment (**Figure 4C**). SCDY1- and mock-treated samples showed limited separation under non-saline conditions but were more clearly separated under 120 mM NaCl, and biological replicates clustered closely within each treatment group.

**Figure 4:**
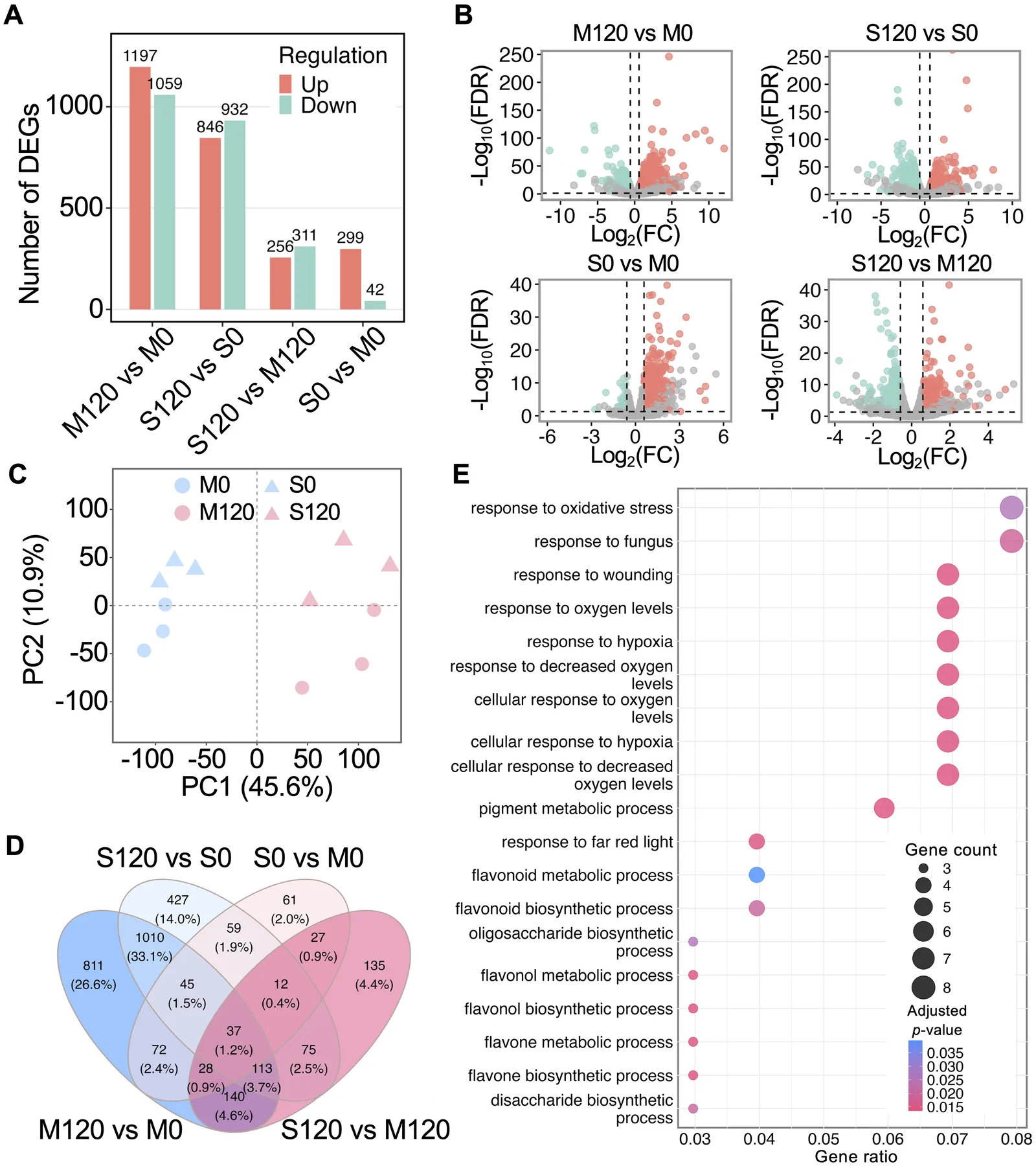
Transcriptome analysis of SCDY1-treated *Arabidopsis* seedlings under moderate salinity stress. **(A)** Numbers of differentially expressed genes (DEGs) identified in four pairwise comparisons: M120 vs M0, S120 vs S0, S120 vs M120, and S0 vs M0. Upregulated and downregulated DEGs are shown separately. **(B)** Volcano plots of DEGs identified in the four pairwise comparisons. Red and blue points indicate upregulated and downregulated genes, respectively, and gray points indicate genes that were not significantly differentially expressed. **(C)** Principal component analysis (PCA) of RNA-seq samples from the four treatment groups. PC1 explained 45.6% of the total variance and primarily separated samples according to salinity treatment, whereas PC2 explained 10.9% of the variance and reflected variation associated with SCDY1 treatment. **(D)** Venn diagram showing overlap among the four pairwise DEG lists. Of 3,052 unique DEGs, 135 were detected only in the S120 vs M120 comparison (**Supplementary Table 9**); 32 of these also belonged to the 478 genes showing a significant SCDY1 inoculation × salinity interaction (**Supplementary Table 8**). **(E)** Gene Ontology enrichment analysis of the 135 comparison-specific DEGs. Oxygen-level- and hypoxia-response terms formed the predominant enrichment theme, whereas responses to wounding, fungus, and oxidative stress were additionally enriched. Dot size indicates gene count, and dot color indicates the adjusted *p* value. Complete results are provided in **Supplementary Table 10**. M0, mock-treated seedlings under non-saline conditions; M120, mock-treated seedlings under 120 mM NaCl; S0, SCDY1-treated seedlings under non-saline conditions; S120, SCDY1-treated seedlings under 120 mM NaCl.

A gene-wise analysis across all four conditions identified 478 genes with a significant SCDY1 inoculation × salinity interaction (FDR < 0.05; **Supplementary Table 8**). Comparison of the four pairwise DEG lists identified 3,052 unique DEGs, including 135 detected only in S120 vs M120 (**Figure 4D**; **Supplementary Table 9**); 32 of these genes (23.7%) also showed a significant interaction (**Supplementary Table 8**). Accordingly, the 135 DEGs were treated as a comparison-specific subset rather than as formal interaction genes and were subjected to GO enrichment analysis. Oxygen-level- and hypoxia-response terms formed the predominant enrichment theme, whereas terms related to responses to wounding, fungus, and oxidative stress were additionally enriched (**Figure 4E**; **Supplementary Table 10**).

### 3.5 SCDY1-responsive gene clusters reveal stress- and root-associated transcriptional modules under salinity stress

To further characterize the biological processes associated with SCDY1-dependent transcriptional changes under salt stress, we performed hierarchical clustering of DEGs identified across the treatment comparisons. The DEGs were classified into 10 clusters according to their expression patterns across the four treatment groups (**Figure 5A**; **Supplementary Table 11**). These clusters revealed distinct transcriptional modules associated with salinity stress and SCDY1 inoculation, suggesting that SCDY1 modulates specific subsets of stress-responsive genes rather than globally altering host gene expression. Among the 10 clusters, Cluster 1 contained a major group of genes enriched for biological processes related to response to water deprivation, response to oxidative stress, and response to jasmonic acid (**Figure 5B**). Cluster 6 was enriched for genes associated with response to oxygen levels, cellular response to oxygen levels, and response to hypoxia (**Figure 5C**). Cluster 9 contained a smaller set of genes enriched for plant-type cell wall organization or biogenesis, plant epidermis development, and root epidermal cell differentiation (**Figure 5D**). These clusters linked SCDY1-responsive transcriptional changes under salinity stress to water stress, oxygen/redox regulation, and root cell wall- or epidermis-related processes.

**Figure 5:**
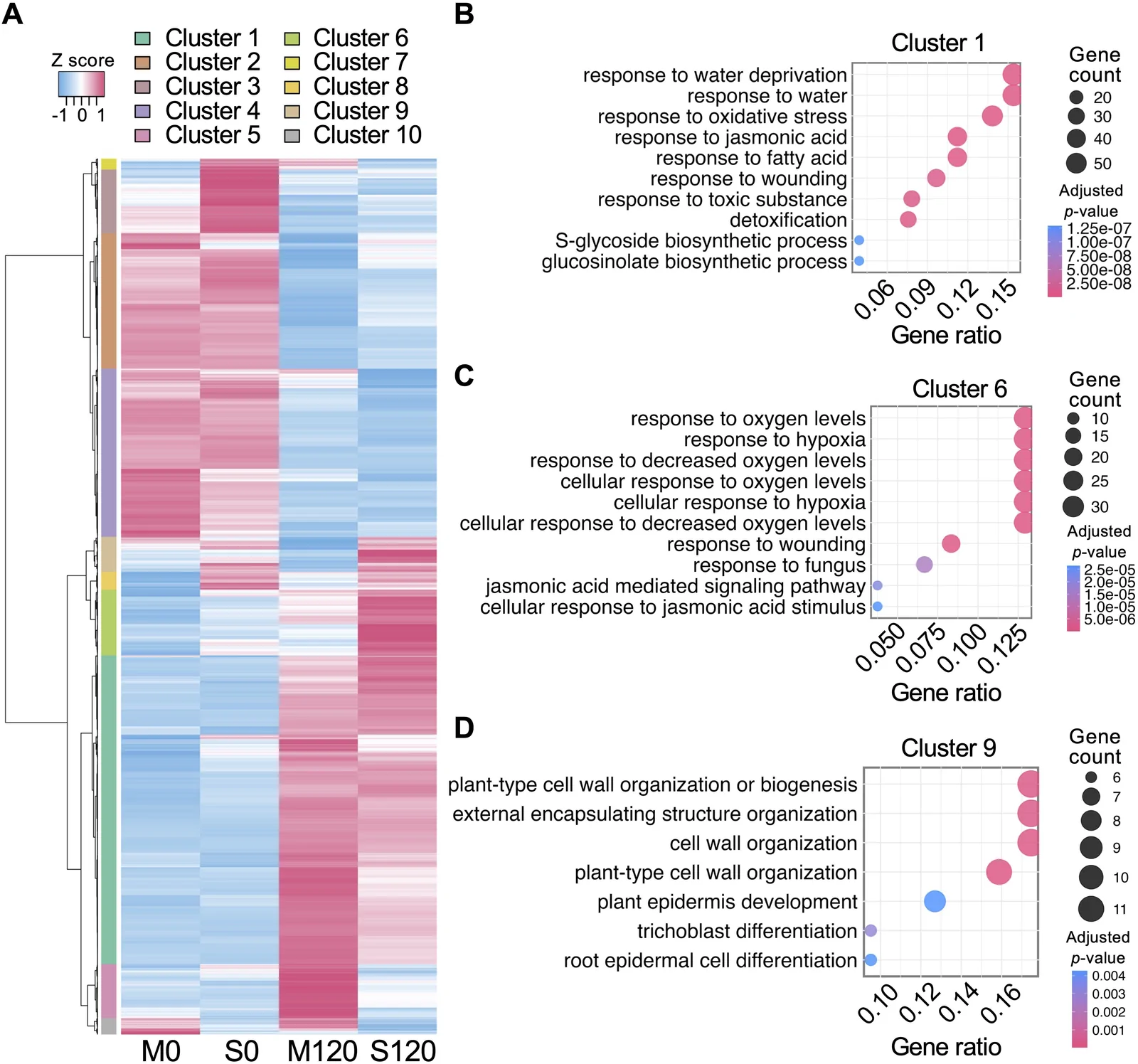
Transcriptional modules associated with SCDY1 treatment and salinity. **(A)** Hierarchical clustering of DEGs identified in at least one of the four pairwise comparisons across M0, S0, M120, and S120. DEGs were classified into 10 clusters based on their expression patterns. Red and blue indicate relatively high and low expression levels, respectively. **(B–D)** Gene Ontology enrichment analyses of representative clusters. Cluster 1 was enriched for responses to water deprivation, oxidative stress, and jasmonic acid **(B)**; cluster 6 was enriched for responses to oxygen levels, hypoxia, and wounding **(C)**; and cluster 9 was enriched for plant-type cell wall organization or biogenesis, plant epidermis development, and root epidermal cell differentiation **(D)**. These enrichments associate the three modules with abiotic-stress signaling, oxygen-status and wound responses, and cell wall- and root epidermis-related processes, respectively. Dot size indicates the number of genes, and dot color indicates the adjusted *p* value. M0, mock-treated seedlings under non-saline conditions; S0, SCDY1-treated seedlings under non-saline conditions; M120, mock-treated seedlings under 120 mM NaCl; S120, SCDY1-treated seedlings under 120 mM NaCl.

We next performed GO enrichment analysis of all 567 DEGs identified in the S120 vs M120 comparison to obtain a broader overview of enriched biological functions. In the biological process category, the strongest enrichment comprised a group of closely related terms associated with responses to oxygen levels and hypoxia. Response to wounding, response to oxidative stress, and response to water were also prominently enriched (**Figure 6A**; **Supplementary Table 12**). In the molecular function category, the strongest enrichments involved oxidoreductase activity acting on peroxide as acceptor, antioxidant activity, and peroxidase activity, whereas structural constituent of cell wall and monooxygenase activity represented additional enriched terms (**Figure 6B**; **Supplementary Table 13**). Together, the broader GO analysis associated the DEG set from the S120 vs M120 comparison primarily with functions related to oxygen status and redox regulation, with additional links to processes related to water stress and the cell wall.

**Figure 6:**
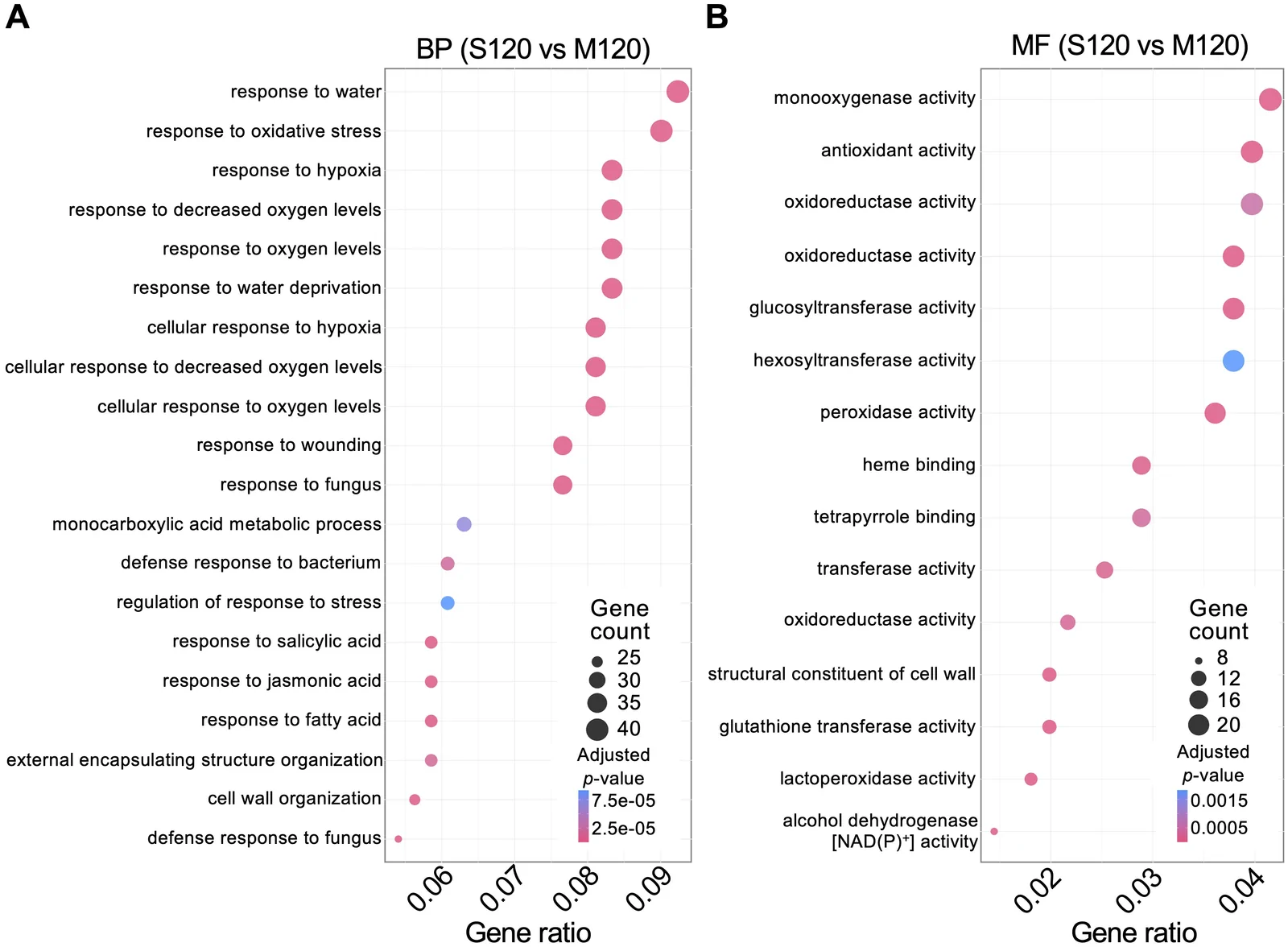
Gene ontology enrichment analysis of DEGs identified in the S120 vs M120 comparison. **(A, B)** Dot plots showing enriched Gene Ontology terms among all 567 DEGs identified in the S120 vs M120 comparison. Biological process terms are shown in **(A)**, and molecular function terms are shown in **(B)**. The biological process enrichment was dominated by closely related terms associated with responses to oxygen levels and hypoxia. Additional prominent terms included response to wounding, response to oxidative stress, and response to water. The strongest molecular function enrichments involved oxidoreductase activity acting on peroxide as acceptor, antioxidant activity, and peroxidase activity. Collectively, these results associate the S120 vs M120 DEG set primarily with processes related to oxygen status and redox regulation. Dot size indicates the number of genes assigned to each GO term, and dot color indicates the adjusted *p* value. S120, SCDY1-treated seedlings under 120 mM NaCl; M120, mock-treated seedlings under 120 mM NaCl. Complete enrichment results for panels **(A)** and **(B)** are provided in **Supplementary Table 12** and **Supplementary Table 13**, respectively. GO term labels in panel **(B)** were abbreviated for readability; their full descriptions are provided in **Supplementary Table 13**.

### 3.6 SCDY1-responsive genes are associated with phenylpropanoid biosynthesis, root-associated responses, and glutathione metabolism

To identify metabolic and signaling pathways associated with SCDY1-responsive transcriptional changes under salt stress, we performed KEGG pathway enrichment analysis using DEGs identified in the S120 vs M120 comparison. KEGG analysis showed enrichment of genes associated with phenylpropanoid biosynthesis, glutathione metabolism, and plant MAPK signaling (**Figure 7**; **Supplementary Table 14**). Among these pathways, phenylpropanoid biosynthesis and glutathione metabolism were most directly related to the cell wall-associated and redox-related responses identified by GO enrichment analysis.

**Figure 7:**
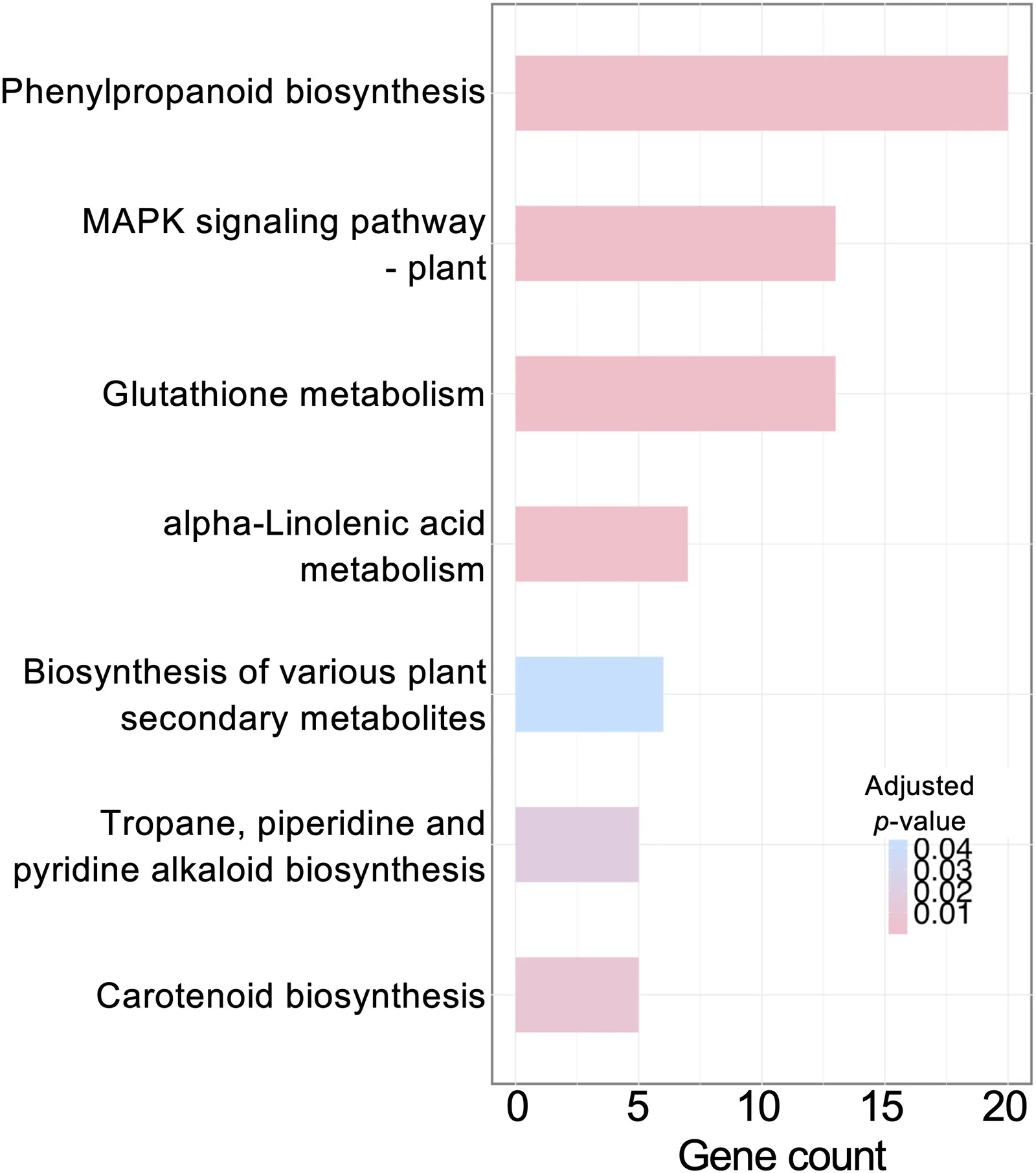
KEGG pathway enrichment analysis of DEGs identified in the S120 vs M120 comparison. Bars indicate the number of DEGs assigned to each enriched KEGG pathway, and color indicates the adjusted *p* value. Enriched pathways included phenylpropanoid biosynthesis, plant MAPK signaling, and glutathione metabolism, associating the S120 vs M120 DEG set with cell wall-related metabolism, stress signaling, and redox-related processes. S120, SCDY1-treated seedlings under 120 mM NaCl; M120, mock-treated seedlings under 120 mM NaCl.

Phenylpropanoid biosynthesis showed a prominent transcriptional signature, with 20 assigned DEGs, including 12 upregulated and 8 downregulated genes in SCDY1-treated plants under salt stress (**Figure 7**; **Supplementary Table 15**). Several class III peroxidase genes, including *AtPRX1*, *PRX2*, *PER7*, *PRX8*, *PRX35*, *PRX37*, *PER39*, *PRX44*, and *PRX73*, were upregulated in SCDY1-treated seedlings. Root hair-specific genes, including *RHS18* and *RHS19*, were also upregulated (**Supplementary Table 15**). Thus, the phenylpropanoid-related DEG set included genes associated with cell wall modification and root epidermal responses.

Genes assigned to glutathione metabolism showed a predominantly downregulated pattern in SCDY1-treated plants under salt stress. Thirteen DEGs were assigned to this pathway, including glutathione S-transferase genes such as *GSTU1*, *GSTU4*, *GSTU7*, *GSTU17*, *GSTU22*, *GSTU24*, *GSTU25*, *GSTF2*, and *GSTF6*, as well as redox-related genes including *DHAR2* and *G6PD3* (**Figure 7**; **Supplementary Table 16**). Stress-responsive genes such as *ERD9* and *ERD11* also showed reduced expression (**Supplementary Table 16**), linking SCDY1 treatment to glutathione- and redox-associated transcriptional responses under salinity stress.

We next performed targeted RT-qPCR analysis of selected genes using the same RNA samples used for RNA-seq analysis. The selected genes included class III peroxidase genes (*PRX44*, *PER39*, *PRX2*, and *PRX35*), root hair- or root epidermis-related genes (*RHS18* and *RHS19*), and glutathione metabolism- or redox-related genes (*GSTF6*, *GSTU24*, *GSTU7*, *DHAR2*, and *ERD9*) (**Figure 8**). These genes were selected based on the RNA-seq and pathway analyses (**Supplementary Tables 15**, **16**). RT-qPCR analysis supported the RNA-seq expression patterns: *PRX44*, *PER39*, *PRX2*, *PRX35*, *RHS18*, and *RHS19* showed increased expression in SCDY1-treated seedlings, whereas *GSTF6, GSTU24, GSTU7, DHAR2*, and *ERD9* showed reduced expression (**Figure 8**). Together, KEGG pathway analysis and targeted RT-qPCR analysis linked SCDY1-responsive transcriptional changes to phenylpropanoid biosynthesis, root hair-related responses, and glutathione-associated redox metabolism under salinity stress.

**Figure 8:**
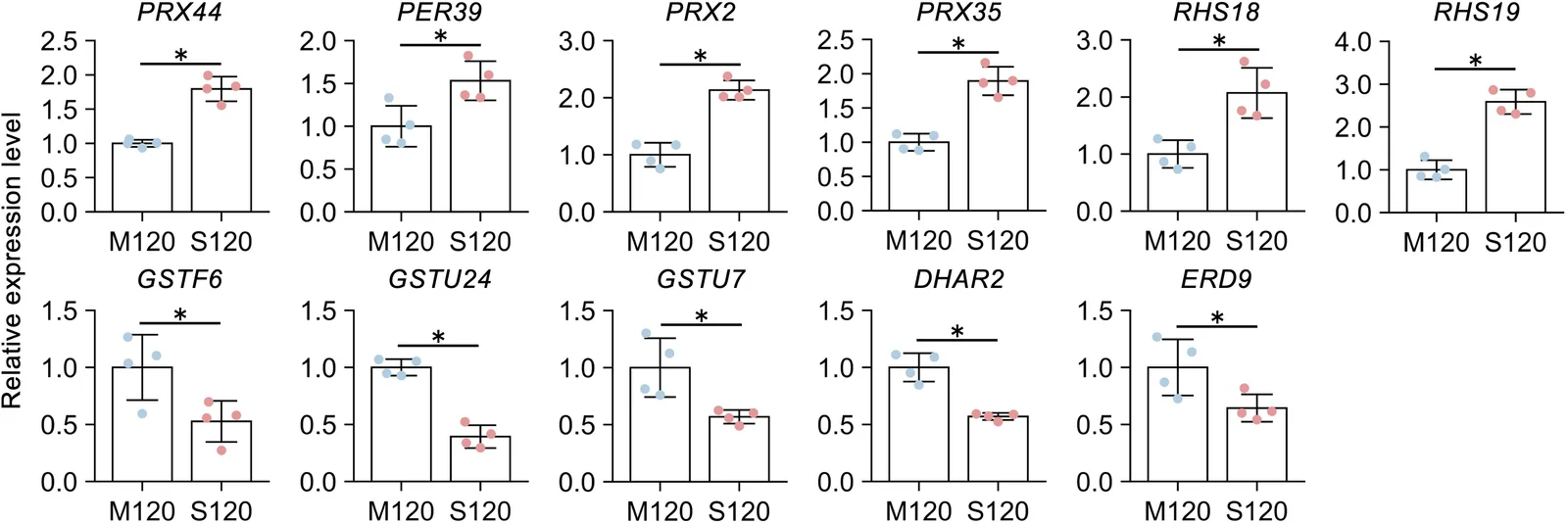
Targeted RT-qPCR analysis of selected SCDY1-responsive genes under salinity stress. Relative expression levels of selected phenylpropanoid- and cell wall-related genes (*PRX44, PER39, PRX2, and PRX35*), root hair- or root epidermis-related genes (*RHS18* and *RHS19*), and glutathione metabolism- or redox-related genes (*GSTF6, GSTU24, GSTU7, DHAR2*, and *ERD9*) were analyzed in mock-treated and SCDY1-treated *Arabidopsis* seedlings grown under 120 mM NaCl. Gene expression levels were quantified by RT-qPCR and normalized to *PP2A* as the reference gene. Expression levels are shown as fold changes relative to the mean expression level of mock-treated seedlings under 120 mM NaCl (M120), which was set to 1 for each gene. Bars represent means ± SD, and individual points represent biological replicates (*n* = 4). Statistical significance between M120 and S120 was assessed using a two-sided Student’s *t*-test. Asterisks indicate significant differences (*p < 0.05). M120, mock-treated seedlings under 120 mM NaCl; S120, SCDY1-treated seedlings under 120 mM NaCl.

### 3.7 SCDY1 treatment enhances root hair-related phenotypes in *Arabidopsis* seedlings

Because transcriptome and targeted RT-qPCR analyses linked SCDY1 treatment to root epidermis- and root hair-related genes, we examined root hair phenotypes under non-saline and saline conditions. Five-day-old seedlings grown under 0 or 120 mM NaCl were inoculated with mock solution or SCDY1, and primary root and root hair phenotypes were analyzed 48 h after inoculation (**Figure 9A**). Primary root length was reduced under 120 mM NaCl but did not differ significantly between mock- and SCDY1-treated seedlings under either NaCl condition (**Figure 9B**), allowing root hair-related phenotypes to be evaluated without detectable treatment-dependent differences in primary root elongation.

**Figure 9:**
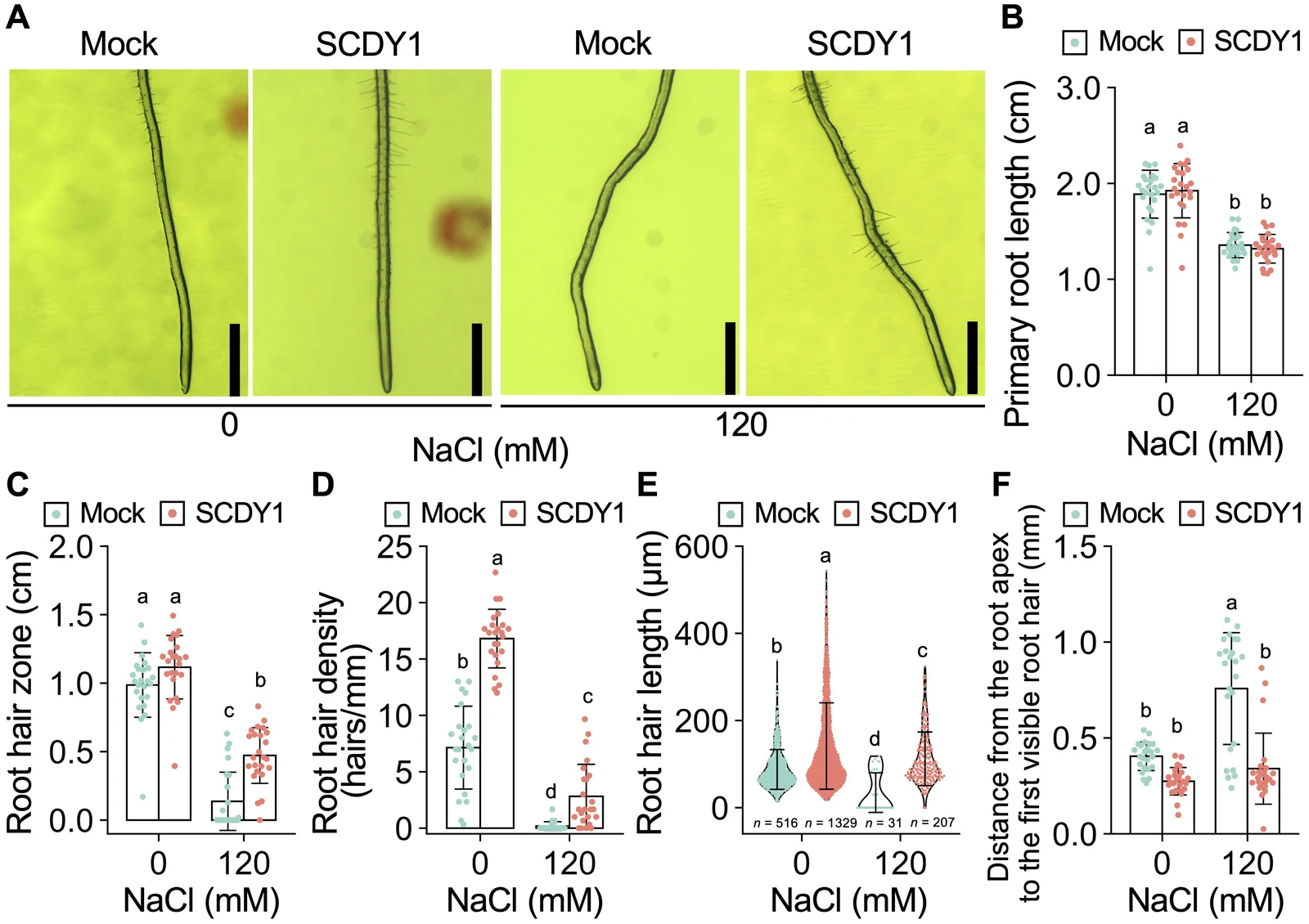
SCDY1 treatment enhances root hair-related phenotypes in *Arabidopsis* seedlings. Five-day-old *Arabidopsis* seedlings grown on 0.5× MS medium with or without 120 mM NaCl were inoculated with mock solution or SCDY1, and primary root and root hair phenotypes were analyzed 48 h after inoculation. **(A)** Representative images of root hairs in mock-treated and SCDY1-treated seedlings under 0 and 120 mM NaCl conditions. Scale bar = 1 mm. **(B)** Primary root length. **(C)** Root hair zone length, measured as the maturation zone formed after inoculation to the last visible root hair. **(D)** Root hair density, quantified in a 3-mm root segment located 2 mm from the primary root tip and expressed as the number of root hairs per millimeter of primary root length. **(E)** Root hair length measured in the same root region. **(F)** Distance from the root apex to the first visible root hair, used as an index of the position of root hair differentiation. For **(B–D, F)**, bars represent mean ± SD, and dots indicate individual biological replicates (*n* = 24). For **(E)**, violin plots show the distribution of root hair length, box plots indicate the median and interquartile range, and dots indicate individual root hair measurements. Sample sizes for **(E)** are indicated below each violin plot. Different letters indicate significant differences among treatment groups based on one-way ANOVA followed by Tukey’s HSD test (*p* < 0.05).

Root hair zone length did not differ significantly between mock- and SCDY1-treated seedlings under 0 mM NaCl, but was significantly greater in SCDY1-treated seedlings than in mock-treated seedlings under 120 mM NaCl (**Figure 9C**). Root hair density and root hair length were significantly higher in SCDY1-treated seedlings than in mock-treated seedlings under both 0 and 120 mM NaCl conditions (**Figures 9D, E**). Although salinity reduced root hair density and root hair length, SCDY1-treated seedlings maintained higher values than mock-treated seedlings under salinity stress. In addition, the distance from the root apex to the first visible root hair was shorter in SCDY1-treated seedlings than in mock-treated seedlings under 120 mM NaCl, whereas no significant difference was detected under 0 mM NaCl (**Figure 9F**). This suggests that SCDY1 treatment is associated with an earlier onset of root hair differentiation under salinity stress. Together, these results show that SCDY1 enhances root hair-related phenotypes and shifts visible root hair formation closer to the root apex without detectable treatment-dependent changes in primary root length, providing phenotypic support for SCDY1-associated root epidermal responses under moderate salinity stress.

## 4 Discussion

Here, we developed a cube-based SynCom screening strategy to identify quinoa-derived bacterial consortia that mitigate salt stress-associated growth inhibition in *Arabidopsis*. By screening 135 quinoa-associated bacterial isolates as 45 defined 9-isolate SynComs, we identified SynCom DY1 (SCDY1) as a taxonomically diverse consortium that promoted *Arabidopsis* growth under moderate salinity stress (**Figures 1**, **3**). A key feature of this screening design is that it preserves structured combinatorial diversity while keeping the number of SynComs within a practical screening scale (**Figure 1**). Compared with random assembly, the cube-based design introduces a defined structure that retains traceability of individual isolates across multiple SynComs. Trait-based approaches prioritize isolates with predefined functional properties, whereas reduced-complexity approaches typically simplify a natural or host-selected microbiota into a tractable representative community (Niu et al., 2017). The cube-based approach instead retains broader combinatorial diversity within a large isolate collection while keeping the number of communities experimentally manageable, thereby allowing potentially beneficial combinations that are not readily predicted from individual isolate traits to be explored. As with any experimentally tractable combinatorial screening strategy, the cube-based design samples only a small fraction of the total combination space. A specific synergistic interaction between two isolates can therefore be missed if the isolates are assigned to different 27-isolate sets or are never included together in the same 9-isolate layer. This is an inherent limitation of the design. At the same time, even within a single 27-isolate set, 4,686,825 (= 27!/[9!18!]) possible nine-isolate combinations exist, and across all 135 isolates, the number exceeds 31 trillion (31,254,974,939,760 = 135!/[9!126!]), making exhaustive screening experimentally unrealistic. The cube-based design reduces this search space to 45 structured SynComs while representing every isolate in three distinct community contexts. This repeated, non-random representation preserves isolate traceability and provides initial information on isolate-level versus combination-dependent activity. Thus, the approach trades exhaustive coverage for a structured and experimentally feasible way to interrogate a large isolate pool. However, the present analysis does not fully distinguish among dominant-isolate, additive, and interaction-dependent contributions within the selected SynCom. SCDY1 exhibited a broad multifunctional profile, including traits related to nutrient mobilization, indole compound production, iron acquisition, cell wall polymer degradation, and bacterial growth under saline conditions (**Figure 2**). Because these traits were distributed among different member isolates, SCDY1 contains complementary functional capacities that may contribute to its ability to mitigate plant growth inhibition under salt stress.

Across the tested salinity range, SCDY1-associated growth promotion in *Arabidopsis* was most clearly observed under 100–120 mM NaCl (**Figure 3**). A significant SCDY1 treatment × NaCl interaction detected by plate-level analysis of primary root length at 187 h provided direct statistical support for a salinity-dependent effect of SCDY1 on root elongation. Together, these findings indicate that SCDY1-associated growth promotion is stress-context dependent rather than constitutive across salinity conditions. Bacterial re-isolation from inoculated seedlings showed that several SCDY1 constituent bacteria were recoverable during the assay period. However, this culture-dependent assay does not establish stable community establishment, persistence, or colonization, nor does it resolve the abundance or spatial distribution of all consortium members. It nevertheless confirmed the presence of recoverable SCDY1-derived bacteria under the conditions in which growth promotion was observed. Because only four of the nine inoculated isolates were recovered by this culture-dependent assay, the composition of the SCDY1 community during the plant assay remains uncertain, and community succession or reduction cannot be excluded. However, failure to recover the other five isolates does not demonstrate their absence, because low-abundance or poorly culturable cells may not have been detected. Accordingly, the observed growth-promoting phenotype should be interpreted as an effect of inoculation with the nine-strain SCDY1 rather than as evidence that all nine members persisted or that the four recovered isolates alone were responsible for the phenotype. Distinguishing among these possibilities will require reduced-community reconstruction, dropout analysis, and quantitative tracking of individual community members. Together, these findings support a model in which SCDY1 promotes *Arabidopsis* growth most effectively under moderate salinity stress, with several constituent bacteria recoverable from the plant assay system.

Host transcriptome analysis provided insight into plant responses associated with SCDY1-mediated growth promotion under salt stress. Salt stress is known to restrict root growth by affecting root meristem activity, auxin distribution, redox status, water relations, and cell expansion (Jiang et al., 2016; Liu et al., 2015). In our dataset, salinity was the dominant driver of transcriptional variation, whereas SCDY1 treatment was associated with a smaller but distinct transcriptional shift (**Figure 4**). Gene-wise interaction analysis identified 478 genes with significant SCDY1 inoculation × salinity interactions, including 32 of the 135 DEGs detected only in the S120 vs M120 pairwise comparison (**Supplementary Tables 8**, **9**). The balanced up- and downregulation in the S120 vs M120 comparison suggests selective modulation rather than general activation of the host salt-stress response. Functional analyses of the pairwise-derived DEG sets associated SCDY1 treatment primarily with oxygen-status- and redox-related processes, with additional links to cell wall- and root epidermis-related processes (**Figures 5**–**8**). Among these responses, the enrichment of GO terms related to cell wall organization, plant epidermis development, and root epidermal cell differentiation was particularly relevant to the root hair-related phenotypes observed in SCDY1-treated seedlings, because root epidermal cells and root hairs are highly plastic structures that respond to nutrient availability, water status, and abiotic stress (Zhang et al., 2023). At 48 h after inoculation, SCDY1 enhanced several root hair traits and shifted visible root hair formation closer to the root apex, without a detectable treatment-dependent difference in primary root length (**Figure 9**), indicating an early root epidermal response rather than a consequence of increased root elongation. Several SCDY1-responsive genes encode class III peroxidases, a protein family associated with ROS metabolism, cell wall modification, and root hair growth (Cosio and Dunand, 2009; Marjamaa et al., 2009; Pacheco et al., 2022; Passardi et al., 2004). Although the RNA-seq, RT-qPCR, and root hair data do not establish causality, they identify redox- and cell wall-related root epidermal responses as candidate links to later growth promotion for future functional testing.

Previous stress-microbiome and SynCom studies provide an important context for the present work. A halophyte-associated microbiome was previously shown to help non-host plants withstand salinity, supporting the idea that stress-adapted plants can harbor microbial resources that improve plant stress performance (Yuan et al., 2016). Similarly, a synthetic bacterial community derived from a desert rhizosphere was shown to confer salt stress resilience to tomato even in the presence of a soil microbiome (Schmitz et al., 2022). In line with these studies, our results support the view that plants adapted to stressful environments can serve as useful sources of beneficial microorganisms. The use of *Arabidopsis* as the validation host was therefore an intentional component of the experimental design rather than a substitute for validation in quinoa. Our objective was to test whether microbial functions associated with a highly salt-tolerant plant could be expressed as measurable benefits in a more salt-sensitive glycophytic host. Because quinoa itself is highly salt tolerant, the use of *Arabidopsis* provides a sensitive and experimentally tractable system for quantitatively resolving microbial effects on plant growth and host responses under salinity stress. Thus, the cross-species assay directly addresses the broader potential of stress-adapted plants as sources of beneficial microorganisms for other plant species.

The distinct contribution of the present work is the cube-based screening framework, which enabled evaluation of a large quinoa-derived isolate collection at a practical scale while preserving the traceability of isolate combinations (**Figure 1**). The present study establishes a controlled screening platform that can now be extended in several directions. Dropout SynCom analysis, reduced SynCom reconstruction, and comparison with selected individual isolates will help define the member isolates or functional groups responsible for SCDY1 activity. Isolate-specific qPCR, amplicon sequencing, or fluorescent labeling will clarify the persistence, spatial distribution, and relative abundance of SCDY1 members on or near plant roots. Further analyses of cell wall modification, ROS distribution, and root hair-related pathways will help test how root epidermal responses contribute to growth promotion. Finally, testing SCDY1 or related quinoa-derived SynComs in additional glycophytic crop species and soil-based systems will be important for evaluating the broader applicability of this approach. Thus, the cube-based SynCom strategy developed here provides a practical starting point for moving from large stress-adapted bacterial collections toward defined microbial communities with testable functions in plant stress performance.

## 5 Conclusions

This study developed a cube-based screening strategy for identifying beneficial SynComs from quinoa-associated bacterial collections. Using this approach, we identified SCDY1 as a taxonomically diverse 9-isolate SynCom with multifunctional plant growth-promoting traits and growth-promoting activity under moderate salinity conditions in *Arabidopsis*. Bacterial re-isolation supported the presence of SCDY1-derived bacteria in the plant assay system. Host transcriptome analysis, targeted RT-qPCR analysis, and root hair phenotyping linked SCDY1 treatment to stress-responsive and redox-associated transcriptional changes as well as enhanced root hair-related epidermal phenotypes, including a shift in visible root hair formation toward the root apex, under moderate salinity stress. These findings show that a SynCom assembled from quinoa-associated bacterial isolates can enhance *Arabidopsis* performance under salinity stress and is associated with complementary microbial traits and modulation of host root-associated stress responses. Together, this study provides a practical framework for moving from large plant-associated bacterial collections to defined SynComs with testable functions in plant performance under salinity stress.

## Data Availability

The datasets generated in this study are available at the INSDC (DDBJ, EMBL, and GenBank) under BioProject accession PRJDB42650 (https://ddbj.nig.ac.jp/search/entry/bioproject/PRJDB42650/).
